# Variable patterns of ectopic mineralization in *Enpp1*^asj-2J^ mice, a model for generalized arterial calcification of infancy

**DOI:** 10.18632/oncotarget.13335

**Published:** 2016-11-14

**Authors:** Sarah Y. Siu, Nathaniel A. Dyment, David W. Rowe, John P. Sundberg, Jouni Uitto, Qiaoli Li

**Affiliations:** ^1^ Department of Dermatology and Cutaneous Biology, The Sidney Kimmel Medical College and The PXE International Center of Excellence in Research and Clinical Care, Thomas Jefferson University, Philadelphia, PA, USA; ^2^ Center for Regenerative Medicine and Skeletal Development, University of Connecticut Health Center, Farmington, CT, USA; ^3^ The Jackson Laboratory, Bar Harbor, ME, USA; ^4^ Jefferson Institute of Molecular Medicine, Thomas Jefferson University, Philadelphia, PA, USA

**Keywords:** ectopic mineralization, generalized arterial calcification of infancy, mouse models, cryohistology, Pathology Section

## Abstract

Generalized arterial calcification of infancy (GACI) is an autosomal recessive disorder characterized by early onset of extensive mineralization of the cardiovascular system. The classical forms of GACI are caused by mutations in the *ENPP1* gene, encoding a membrane-bound pyrophosphatase/phosphodiesterase that hydrolyzes ATP to AMP and inorganic pyrophosphate. The *asj-2J* mouse harboring a spontaneous mutation in the *Enpp1* gene has been characterized as a model for GACI. These mutant mice develop ectopic mineralization in skin and vascular connective tissues as well as in cartilage and collagen-rich tendons and ligaments. This study examined in detail the temporal ectopic mineralization phenotype of connective tissues in this mouse model, utilizing a novel cryo-histological method that does not require decalcification of bones. The wild type, heterozygous, and homozygous mice were administered fluorescent mineralization labels at 4 weeks (calcein), 10 weeks (alizarin complexone), and 11 weeks of age (demeclocycline). Twenty-four hours later, outer ears, muzzle skin, trachea, aorta, shoulders, and vertebrae were collected from these mice and examined for progression of mineralization. The results revealed differential timeline for disease initiation and progression in various tissues of this mouse model. It also highlights the advantages of cryo-histological fluorescent imaging technique to study mineral deposition in mouse models of ectopic mineralization disorders.

## INTRODUCTION

Ectopic mineralization, characterized by deposition of hydroxyapatite on soft connective tissues, is commonly associated with arteriosclerosis, diabetes, chronic renal disease, inflammatory connective tissue diseases, and a number of genetic disorders [1]. Generalized arterial calcification of infancy (GACI) is a heritable ectopic mineralization disorder in humans in which the arterial blood vessels are severely affected. The disease is often diagnosed by prenatal ultrasound or shortly after birth with widespread mineralization in the arteries. The disease presents clinically with heart failure, respiratory distress, hypertension, cyanosis, and cardiomegaly. The majority of affected individuals die within the first year of life from cardiovascular collapse caused by mineralization of blood vessels. GACI is inherited in an autosomal recessive fashion. The classic form of GACI is caused by mutations in the *ENPP1* gene, which encodes ectonucleotide pyrophosphatase/phosphodiesterase 1 (ENPP1), an enzyme that hydrolyses ATP to AMP and inorganic pyrophosphate (PPi) [2, 3]. Under physiologic conditions, PPi serves as a powerful anti-mineralization factor preventing ectopic mineralization. In GACI, as a result of reduced ENPP1 activity, the ratio of inorganic phosphate (Pi) to PPi increases, which creates a pro-mineralization environment and allows ectopic tissue mineralization to ensue. There is currently no effective treatment for GACI.

Mouse models for GACI have been helpful in identifying critical pathways involved and exploring potential treatments for this, currently intractable, life-threatening disorder. One of them, the *Enpp1^asj-2J ^*mouse (referred to hereon as *asj-2J* mouse), arose spontaneously in a large-scale production colony of BALB/cJ mice at The Jackson Laboratory [4]. These mutant mice were allelic to previously characterized *asj* mouse harboring a p.V246D mutation in the *Enpp1* gene [5]. These mice develop abnormal forepaw position due to stiffening of the joints, a phenotype known as asj (“ages with stiffened joints”). The *asj-2J* mice carry a large, 40,035 bp deletion from intron 1 to 3’-untranslated region of the *Enpp1* gene, eliminating the majority of the gene sequence, coupled with a 74 bp insertion. Previous studies in *asj-2J* mice focused on characterization of ectopic mineralization in soft connective tissues including the skin and the cardiovascular system, by a combination of histopathology with calcium-specific stains, direct chemical assay of calcium, and microcomputed tomography [4]. Recently, ectopic mineralization of cartilage and periarticular tendons and ligaments were also examined in these mice. A standard histopathologic approach combined with a novel cryo-sectioning technique of bones without decalcification demonstrated extensive mineralization of cartilage as well as periarticular tendons and ligaments [6]. However, the temporal growth of mineral deposits in these ectopic sites could not be analyzed by the traditional techniques of histology.

In this study, we characterized the *asj-2J* mouse using fluorescent mineralization labels at different time points of development. We identified various mineralization patterns at ectopic sites of soft connective tissues as well as cartilage and periarticular tendons and ligaments.

## RESULTS

### Experimental design

Previous studies demonstrated that feeding *asj-2J* mice with an “acceleration diet”, enriched in phosphate (2x) and reduced in magnesium content (20%) compared to standard rodent diet, resulted in acceleration of the ectopic mineralization in soft connective tissues [4]. In this study, we placed *Enpp1^+/+^* (*n* = 14), *Enpp1^+/asj-2J ^*(*n* = 16), and *Enpp1^asj-2J^* mice (*n* = 16) on the acceleration diet to shorten the time required for development of the ectopic mineralization phenotype. In addition, these mice received injections of fluorescent labels at three different time intervals, allowing the progression of ectopic mineralization in soft tissues to be examined in the same animals. Specifically, the mice were administered mineralization labels at 4 weeks (calcein; green), 10 weeks (alizarin complexone; red), and 11 weeks of age (demeclocycline; yellow). Mineralization labels given at these time points captured the formation of newly formed mineral deposits. Mice were sacrificed 24 hours after the last injection of demeclocycline and examined for ectopic mineralization in comparison to wild type and heterozygote littermates. A cryo-histological method was used to obtain frozen sections of mineralized tissues for analysis [7-9]. The non-decalcified histology not only identifies areas of mineral accumulation in these tissues, but also maintains the fluorescent signals from mineralization lines to indicate the time at which lesions develop. In addition, the cryosections retain enzymatic activities for fluorescent tartrate-resistant acid phosphate (TRAP) and alkaline phosphatase (AP) assays. Multi-round imaging analysis included Calcein Blue for mineral deposition, AP as an indicator of osteoblasts and bone formation, TRAP as a marker for osteoclasts and bone resorption [10], and Toluidine Blue O for anatomic structure.

### Histopathologic evaluation of ectopic soft tissue mineralization

Toluidine Blue O staining of cryosections revealed ectopic mineralization in a number of tissues including vibrissae of the muzzle skin, outer ear, aorta, trachea, vertebra, and shoulder in *asj-2J* mice. The ectopic mineralization of these tissues serves as a hallmark of the overall mineralization process in the *asj-2J* mice. No mineralization in these tissues was noted in wild type and *asj-2J* heterozygous mice, consistent with previous studies on *asj-2J* mice [4]. When examining the timeline of mineralization in these tissues, differential patterns were observed.

### Differential patters of ectopic mineralization on soft connective tissues

#### Dermal sheath of vibrissae

**Figure 1:**
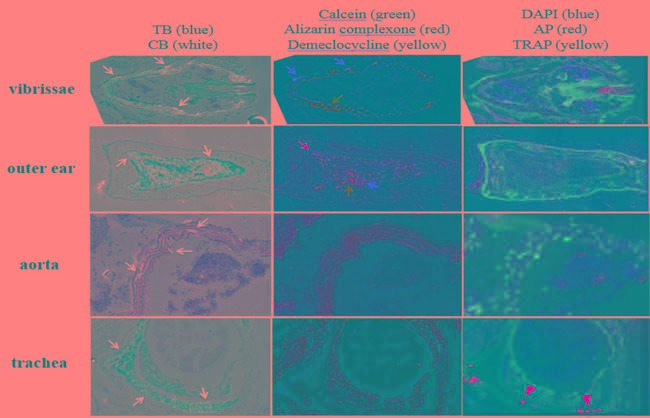
Variable ectopic mineralization patterns in dermal sheath of vibrissae, outer ear, aorta, and trachea of *asj-2J* mice Left panel: Ectopic mineralization is noted in the dermal sheath of vibrissae, elastic cartilage of outer ear, aorta, and hyaline cartilage of trachea (white arrow). Middle panel: Green (calcein, green arrow) and red (alizarin complexone, red arrow) fluorescent labels are noted in vibrissae, when administered at 4 and 10 weeks, respectively. Green (calcein, green arrow), red (alizarin complexone, red arrow), and yellow (demeclocycline, yellow arrow) fluorescent labels are noted in outer ear, when administered at 4, 10, and 11 weeks, respectively. No mineralization labels are found in aorta and trachea. Right panel: Positive TRAP activity is found in trachea only (yellow arrowhead). TB, Toludine Blue O which stains the anatomic structure of tissues. CB, Calcein Blue which stains accumulated minerals. DAPI stains nuclei blue. AP, alkaline phosphatase activity of osteoblasts (red). TRAP, tartrate-resistant acid phosphatase activity of osteoclasts (yellow).

There is extensive ectopic mineralization in the connective tissue capsule of the dermal sheath surrounding vibrissae in muzzle skin of *asj-2J* mice (Figure 1, left panel). Both green (calcein) and red (alizarin) fluorescent labels are seen in the homozygous mice. The majority of the mineralization labeling is green, which was given at 4 weeks of age. A thin layer of red label, which was administered at 10 weeks of age, was found on either side of the green labeling. These mineralization labels demonstrate an organized, outward progression of deposition in the dermal sheath of vibrissae. No yellow (demeclocycline) labeling is evident, which was given at 11 weeks of age (Figure 1, middle panel). These results suggest that most of the mineral deposition in vibrissae occurs around 4 weeks, declines toward the 10 week mark, and is diminished at 11 weeks of age. TRAP and AP activities are entirely negative (Figure 1, right panel), suggesting that ectopic mineralization process was inactive in the muzzle skin when analyzed at 11 weeks.

#### Elastic cartilage

There is evidence of mineralization in the elastic cartilage of outer ear of the *asj-2J* mice (Figure 1, left panel). Green, red and yellow fluorescent labels are found, indicating active mineral deposition at 4, 10 and 11 weeks of age (Figure 1, middle panel). These fluorescent labels are highly disorganized in the ear, showing a very different pattern of mineral formation compared to the dermal sheath of vibrissae. TRAP and AP signals are absent (Figure 1, right panel).

#### Aorta and hyaline cartilage

The aorta and trachea of *asj-2J* mice also exhibit mineral deposition (Figure 1, left panel). No fluorescent labels correspond to the mineral deposits, suggesting that mineralization in the aorta and hyaline cartilage of the trachea occurred prior to the age of 4 weeks and remained stable until 11 weeks of age (Figure 1, middle panel). TRAP and AP activities are negative in the aorta, while positive TRAP activities in trachea signify osteoclast activity and bone resorption at the outer edge of the trachea (Figure 1, right panel).

#### Musculoskeletal soft connective tissues

**Figure 2:**
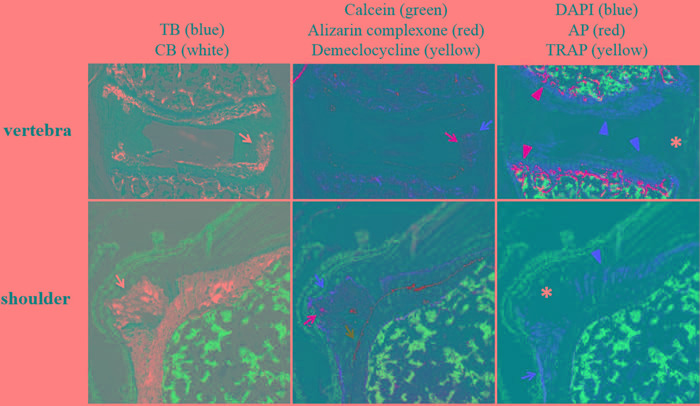
Ectopic mineralization patterns in vertebra and shoulder of *asj-2J* mice Left panel: TB/CB stains identify ectopic mineralization in the annulus of vertebral disc and supraspinatus tendon of the shoulder (white arrow). Middle panel: Red (alizarin complexone, red arrow) and yellow (demeclocycline, yellow arrow) fluorescent labels are noted in the annulus of vertebral disc, when administered at 10 and 11 weeks, respectively. Green (calcein, green arrow), red (alizarin complexone, red arrow), and yellow (demeclocycline, yellow arrow) fluorescent labels are noted in the supraspinatus tendon of the shoulder, when administered at 4, 10, and 11 weeks, respectively. Right panel: Positive AP (red arrowhead) and TRAP activities (yellow arrowhead) are present in the growth plate of vertebra. Organized AP activity (red arrowhead) is also present in the normal enthesis of the shoulder which is mineralizing, while disorganized AP activity (red arrow) is seen in the lateral region of the ectopic mineralization site. Regions of ectopic mineralization in the vertebra and shoulder are negative for AP, TRAP, and cells (asterisk). TB, Toludine Blue O which stains the anatomic structure of tissues. CB, Calcein Blue which stains accumulated minerals. DAPI stains nuclei blue. AP, alkaline phosphatase activity of osteoblasts (red). TRAP, tartrate-resistant acid phosphatase activity of osteoclasts (yellow).

The annulus in the intervertebral disc of *asj-2J* mice reveals mineral accumulation in regions that contain a soft ligamentous tissue (Figure 2, left panel). Ten and 11-week labels are present in islands of mineralization within regions of soft tissue (Figure 2, middle panel). Green label was negative, suggesting that ectopic mineralization occurred between 4 and 11 weeks of age. Despite the intense mineralizing activity, the barrier between fibrocartilage and underlying bone marrow is maintained even though there is high osteoblast and osteoclast activities in the vertebral body as part of the normal mineralization process (Figure 2, right panel). Interestingly, there is a region in the annulus (* in Figure 2, right panel) that consists of mineralized matrix that is devoid of cells.

Shoulders of the *asj-2J* mice have abnormal mineralization in the lateral humeral head adjacent to the enthesis of the supraspinatus tendon (Figure 2, left panel) with clearly demarcated green, red, and yellow fluorescent labels progressing in an outward fashion (Figure 2, middle panel). The calcein label at 4 weeks of age is organized normally, identifying the tidemark in the enthesis as well as the tidemark in the lateral humeral head. However, disorganized mineralized cartilage formed superior to the 4 week calcein label, resulting in a large nodule of ectopic mineralization by 11 weeks of age. The site consists of an area of active mineralization with strong AP activity (red arrow in Figure 2, right panel). This region is disorganized though unlike the adjacent enthesis (red arrowhead in Figure 2, right panel). Similar to the vertebrae, there was also an area of disorganized mineralized matrix that was devoid of cells and AP activity (* in Figure 2, right panel).

## DISCUSSION

This study utilizes a novel cryo-histological fluorescent imaging technique to allow consistent sectioning of mineralized tissues and bones without decalcification [9]. This cryo-histological imaging approach is a paradigm for efficient phenotyping of mineralized tissues. First, a novel method of tape-stabilized cryo-sectioning was utilized to preserve the morphology of mineralized tissues. These sections are then adhered rigidly to glass slides and imaged repeatedly over several rounds of staining. The resultant images are then aligned either manually or *via* computer software to yield composite stacks of several layered images. This allows for co-localization of numerous molecular signals to specific cells within a given section.

There are many advantages to studying mineralization processes with this technique. The standard way to observe mineral deposition in mice requires euthanizing multiple mice at different time points and then comparing the degree of mineralization between different age groups. This requires many animals, which can be difficult when the desired phenotype is a challenge to breed as in the case with *Enpp1^asj-2J^* mice due to stiffened joint phenotype causing limited movement. Comparison is also limited to widely spaced time-points, since differences in deposition must be visually distinctive. The technique of monitoring mineral formation with fluorescent dyes decreases the number of animals used and allows investigation of temporal mineralization in the same animal over time. This protocol can serve as a platform for high-throughput phenotyping of musculoskeletal tissues in GACI and other related mineralization disorders.

One interesting characteristic of the ectopic mineralization in the *asj-2J* mice, which is not found in other genetic or trauma-induced models of ectopic mineralization [8, 11], is that the sites contain regions of disorganized mineral that is completely devoid of cells (* in Figure 2). These regions of dead mineral are usually surrounding by ectopic mineralized cartilage. Therefore, it is likely that the cells died following the deposition of the mineral. This feature is unlike a traditional osteophyte that originates in cartilage or fibrocartilage, matures to mineralized cartilage, and then is replaced by bone. The ectopic mineralization sites seen in the *asj-2J* mice are not replaced by bone. Instead, some of the cells within the mineralized cartilage die.

The methodology utilized in this study provides critical information with a timeline for disease initiation and progression in different tissues. In the *Enpp1^asj-2J^* mice, ectopic mineralization pattern varied greatly between tissues. While deposition was completed in the aorta and trachea at 4 weeks, it was still active in the outer ear, vibrissae, as well as in cartilage and tendon and ligaments in shoulder and vertebrae. This technique helps refine our understanding of the approximate age of the onset of mineralization in various tissues and their progression. For treatment purposes, it is essential to know the timeline of disease initiation in order to determine the appropriate time that treatment should be started. Our data show that mineralization in the aorta and trachea preceded the calcein label given at 4 weeks, indicated by calcium deposits that lack green fluorescent labeling of calcein. Therefore, in order to prevent mineral deposition in these tissues, interventions would conceivably begin before 4 weeks of age. The presence of 4 and 10 week fluorescent labels and lack of 11 week labels in the mineral deposits of the vibrissae, show the mineralization in muzzle skin markedly slowed down or ceased between the 10 and 11 weeks. The ear and shoulder continue to take-up the label at 11 weeks, indicating that mineralization is an on-going process in these tissues. This approach can be used to determine critical time points of disease progression in other calcification disorders as well. The ability to identify initiation of calcium deposition may indicate earlier start times for preventative treatment.

## MATERIALS AND METHODS

### Animals and diet

*Enpp1^asj-2J^* mice (BALB/cJ-*Enpp1^asj-2J^/*GrsrJ*,* stock No: 0191070) were obtained from The Jackson Laboratory (Bar Harbor, ME). *Enpp1^+/+^* as well as heterozygous and homozygous *asj-2J* mutant mice were generated from heterozygous matings. Mice were genotyped and maintained in a climate-controlled environment and fed an ‘acceleration diet’ (Rodent diet TD.00442, Harlan Teklad, Madison, WI), which we previously showed to accelerate the ectopic mineralization in *asj-2J* mice [4]; this diet is enriched in phosphorus (2x) and has reduced magnesium (20%) content. Mice were euthanized by CO_2_ asyphyxiation. All animal experiments were approved by Institutional Animal Care and Use Committee of Thomas Jefferson University. Proper handling and care were followed according to the Animal Welfare Policies of the Public Health Service of the USA.

### Mineralization labeling

Intraperitoneal (IP) injections of calcein (6 mg/kg), alizarin complexone (30 mg/kg) and demeclocycline (60 mg/kg) (Sigma-Aldrich, St. Louis, MO) made up in 2% NaHCO_3_ (pH = 7.4) were delivered to mice to view ectopic mineralization. Calcein was delivered at 4 weeks, alizarin complexone at 10 weeks and demeclocycline at 11 weeks. One day after demeclocycline injection, mice were euthanized and tissues collected for analysis.

### Cryo-histological analysis of bone and cartilage

Ear, muzzle skin containing vibrissae, trachea, aorta, shoulder, and lumbar vertebrae L4-L6 from euthanized mice were fixed in 10% phosphate-buffered formalin for 2 days, transferred to 30% sucrose in PBS overnight, and then embedded in Shandon Cryomatrix (Thermo Fisher Scientific, Waltham, MA). The shoulder was cut in sagittal and coronal planes to investigate the supraspinatus tendon, joint space, and articular cartilage. The lumbar vertebrae were cut in coronal plane to investigate fibrocartilage in the intervertebral discs. All sections were made from non-decalcified joints using cryofilm IIC tape (Section-Lab Co., Hiroshima, Japan), which maintains morphology of mineralized sections. The taped sections were glued to barcoded microscope slides, tissue side up, using UV adhesive glue (Norland Optical Adhesive 63, Norland Products Inc., Cranbury, NJ) and rehydrated prior to staining and imaging [7-9].

### Staining and imaging

Staining and imaging were performed at the University of Connecticut, as previously described [6, 12]. In brief, each section was stained up to 5 times. Tissue adherence to cryofilm tape allows for a coverslip to be removed for multiple rounds of imaging and staining without damaging the tissue. The order of imaging included: 1) fluorescent mineralization labels, 2) Calcein Blue staining, 3) TRAP staining, 4) AP staining, and 5) Toluidine Blue O staining. Slides were stained with Calcein Blue to label mineral deposition. TRAP staining identified by ELF-97 substrate (Life Tech, Grand Island, NY) is a marker for osteoclasts and bone resorption. AP staining identified by Fast red (Sigma Aldrich, St. Louis, MO) is an indicator of bone formation. Lastly, slides were stained with Toluidine Blue O and re-imaged. Epifluorescent and brightfield imaging were performed on the Zeiss Axio Scan. Z1 with chroma filters for each distinct fluorophore (Carl Zeiss Microscopy GmbH, Jena, Germany).

## Abbreviations

GACI: generalized arterial calcification of infancy
asj-2J: ages with stiffened joints - 2 Jackson
TB: Toluidine Blue O
CB: Calcein Blue
AP: alkaline phosphatase
TRAP: tartrate-resistant acid phosphatase
PPi: inorganic pyrophosphate
Pi: inorganic phosphate.
